# Editorial: Muscle Mechanics, Extracellular Matrix, Afferentation, Structural, and Neurological Coupling and Coordination in Health and Disease

**DOI:** 10.3389/fphys.2021.802202

**Published:** 2021-12-06

**Authors:** Can A. Yucesoy, Eva Pontén, Francisco J. Valero-Cuevas, Mark Smeulders, Ciaran Knut Simms

**Affiliations:** ^1^Institute of Biomedical Engineering, Bogaziçi University, Istanbul, Turkey; ^2^Karolinska Institutet, Stockholm, Sweden; ^3^University of Southern California, Los Angeles, CA, United States; ^4^Zuyderland Medical Centre, Sittard, Netherlands; ^5^Trinity College Dublin, Dublin, Ireland

**Keywords:** muscle mechanics, extracellular matrix, connective tissue, neuromusculoskeletal interaction, adaptation, pathology, force production - transmission phenomena

There is a growing emphasis on the importance of muscle extracellular matrix on muscular mechanics and specifically on the effects of the interaction of the extracellular matrix (ECM) and the contractile apparatus. Accordingly, investigation of the role of muscle extracellular matrix and other myofascial tissues continuous with the epimysium on muscle force production was one of the main aims of this Research Topic. From the muscle function viewpoint, the structure and role of intramuscular connective tissue was discussed in a comprehensive review (Purslow). This work placed an emphasis on the shearing of intramuscular connective tissues as the mechanism coordinating forces and displacements between adjacent muscle cells and indicated the role of this in myofascial force transmission (e.g., Huijing, 2009; Yucesoy, 2010). With the aforementioned mechanical interaction accounted for as a key determinant, experimental, and computational quantification assessments for muscle fiber direction length changes or direct sarcomere length changes were also an aim for this Research Topic. An experimental assessment using multi-photon excitation microscopy in mouse tibialis anterior muscle surgically dissected from the tibia and surrounding muscles showed that in isometrically activated muscle the sarcomeres re-organize their lengths into a more uniform pattern over time, whereas in the passive state their length non-uniformity remained the same (Moo and Herzog). Sarcomere lengths in isolated mice soleus muscle *in vivo* were quantified using a force microscope by minimally imaging 20 continuous sarcomere segments, which were used for length measurements (Tsai et al.). Samples from animals exposed to different durations of ischemic stroke suggested that sarcomere additions were absent, with implications for muscle spasticity and/or joint contracture that may follow stroke. Magnetic resonance imaging (MRI) is a powerful tool (Figure 1) to quantify local muscle tissue displacements and strains (e.g., Blemker et al., 2007; Pamuk and Yucesoy, 2015) and tissue structure (e.g., Sinha et al., 2006), while along with continuum-based computational modeling (e.g., Böl and Reese, 2008; Yucesoy and Huijing, 2012) and novel modeling concepts and approaches (see below), these techniques can allow the development of new phenomena (e.g., Cankaya et al., 2021), addressing clinical problems (Westman et al., 2019; Marty and Carlier, 2020; Ramasamy et al.) and understanding mechanisms of treatments (Yucesoy and Huijing, 2009; Turkoglu and Yucesoy, 2016). A review (Sinha et al.) focused on several aspects of advanced MRI analyses, diffusion tensor imaging (DTI), and modeling to quantify muscle tissue strain rates, strains, and fiber orientations to study the effects of aging and disuse-related remodeling of the extracellular matrix on force transmission in the human musculoskeletal system. The authors indicated, in collaboration with Purslow, shearing in the endomysium as a marker for myofascial force transmission, and muscle fiber shear strains for that purpose were quantified recently in human muscle *in vivo* (Pamuk et al., 2016). Although, earlier Street (1983) and recently others (e.g., Purslow; Malis et al.) referred to the mechanical role played by the multimolecular trans-sarcolemmal connections between the endomysium and muscle fibers as lateral force transmission, the term lateral, may not be quite accurate as it suggests force is transmitted perpendicularly to the muscle fiber surface. Several studies report sarcomere length heterogeneity (Pappas et al., 2002; Lichtwark et al., 2018; Moo et al.), which according to myofascial force transmission phenomena is ascribed to myofascial loads (Yucesoy, 2010), i.e., forces acting locally along the muscle fiber originating (i) intramuscularly from the ECM and sarcomeres in neighboring muscle fibers and (ii) epimuscularly from muscle connections to surrounding muscles and other tissues. These forces can affect the mechanical equilibrium determining sarcomere lengths and are aligned with the sarcomeres rather than being normal to them. The principles of this mechanism were studied as a part of this Research Topic using finite element modeling and by comparing isolated muscle with muscle modeled within the framework of its surrounding tissues (Pamuk et al.). The results exemplified heterogeneity of muscle fiber direction length changes occurring in context with myofascial loads acting along the muscle fibers. Two of the three cases studied were designed to represent principles of previous MRI and DTI-based experiments conducted in humans, *in vivo* (Pamuk et al., 2016; Karakuzu et al., 2017). They demonstrate how a passively lengthened muscle can also have shortened parts and how an isometric contracting muscle can also have lengthened parts along the muscle fascicles. In another study *in vivo*, published as a part of this Research Topic, the effects of passive ankle dorsiflexion imposed by an isokinetic dynamometer on human semimembranosus muscle were assessed using high-resolution ultrasound (Wilke and Tenberg, 2020). The authors reported muscle tissue displacement in the dorsal thigh, despite the fact that knee angle was kept constant, and explained this with myofascial force transmission across the knee joint. Such mechanical interaction via the fascial system (Adstrum et al., 2017; Stecco et al., 2018; Wilke et al., 2018) between muscles within the same limb segment (e.g., Huijing et al., 2011; Marinho et al., 2017; Ateş et al., 2018a) and even across segments, e.g., in the gastrocnemius after an imposed anterior pelvic tilt (Cruz-Montecinos et al., 2015) have been reported in numerous studies. In fresh postmortem human subjects, strain mechanisms in lower limb deep fascia induced by passive knee movement were studied using motion analysis and digital image correlation techniques (Sednieva et al.). Shedding light on fascia/muscle interactions, for anterior and lateral fascia, tension, and for the iliotibial tract, dependent knee movement, tension, or pure shear mechanisms were observed in extended or flexed positions, respectively. Based on previous evidence reported on coupling between fascia and skeletal muscle shown in fundamental/animal studies (Huijing, 1999; Huijing and Jaspers, 2005; Stecco et al., 2013; Wilke et al., 2018), using ultrasound (Bojsen-Møller et al., 2010; Tian et al., 2012; Le Sant et al., 2017; Ateş et al., 2018a), using MRI (Huijing et al., 2011; Pamuk et al., 2016; Karakuzu et al., 2017), and intraoperative work (Smeulders et al., 2004, 2005; Smeulders and Kreulen, 2007; de Bruin et al., 2014; Kaya et al., 2019, 2020). Zullo et al. reviewed the functional effects of aging on the neuromusculoskeletal system. They discussed occurrence of loss of effectiveness in the locomotory apparatus, due to the molecular and cellular changes occurring in the myofascia, the skeletal muscle tissue, the nervous system, and their structural and functional coupling.

**Figure 1:**
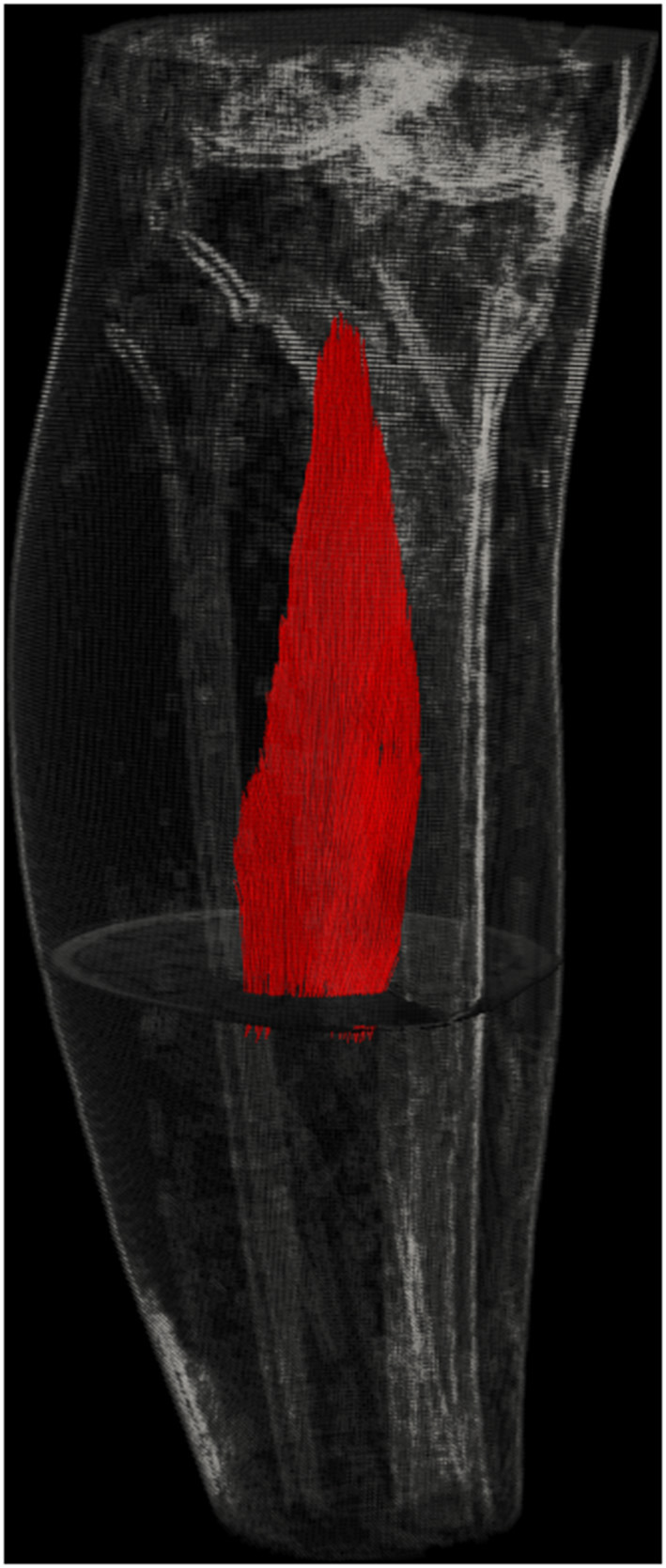
Visualization of human muscle using MRI and DTI. Utilization of advanced imaging techniques offers major potential for analysis of structure as well as local muscle length and shape changes for human muscles *in vivo*.

## Novel Computational Approaches for Different Scales of Neuro-Musculo-Skeletal Mechanics

Understanding the individual contributions of ECM and skeletal muscle fibers to overall passive muscle stiffness presents a particular challenge; Wheatley has used a combination of experimental and computational approaches to show that biaxial tissue testing can provide additional insight over uniaxial test results, and further demonstrate how ECM collagen helps to determine passive muscle stiffness. Klotz et al. discuss fundamental challenges with evaluating detailed constitutive models of skeletal muscle as internal variables are difficult to experimentally quantify, and model predictions at the local muscle level are difficult to verify. As such, these detailed computational models might be considered as philosophical explorations, and their main utility may be to guide the planning of future experimental works. Wakeling et al. and Ryan et al. address energy storage in skeletal muscle, how shape changes in the muscle result in energy storage even without overall length changes, and how some contractile energy is lost in overcoming passive resistance to longitudinal contraction and accompanying lateral expansion due to the largely incompressible nature of the tissue. Ross et al. extend the work of Wakeling et al. and Ryan et al. to dynamic contractions and show how relative energy storage in the aponeurosis increases compared to energy storage in the ECM. Taken together, these papers substantially strengthen our understanding of muscle mechanics.

## Clinical Implications: Mechanisms of Pathological Conditions and Treatment Techniques With a Special Emphasis on Spastic Cerebral Palsy

Cerebral palsy (CP) is defined as decreased motor control due to an injury to the developing brain, before evolvement of mature gait or fine motor skills. Motor control is simplified, and children use simpler muscle synergies while walking compared to typically developed (TD) children. However, treatment for gait improvement only marginally changes muscle synergies (Bekius et al.). Typically, the children are born hypotonic, with a normal range of motion of the joints, but will eventually develop contractures of the joints due to both spasticity and shortening of the musculotendinous complex (Hedberg-Graff et al., 2019; Lindén et al., 2019). At the age of about 9, 3D ultrasound of the medial gastrocnemius muscle and the Achilles tendon has showed that in CP the tendon is relatively longer compared to in TD children, and related to a larger stiffness of the muscle in CP (Weide et al.). Muscle shortening in conjunction with spasticity will force the ankle into flexion, and the heel does not reach the floor when walking, i.e., the children walk with equinus. Proper understanding of the mechanical effect of overactive plantar flexors is critical to clinical diagnosis and treatment, but it can be underestimated if the Achilles tendon moment arm is overestimated by assuming a straight line of action (instead of its actual curved path) (Harkness-Armstrong et al.). One way of diminishing the spasticity is selective dorsal rhizotomy, which by instrumented gait analysis has shown short-term diminishing effects on so-called quasi-stiffness (i.e., the slope of the ankle moment vs. the ankle angle plot) of the calf muscles (Ates et al.). Another local and temporary way of diminishing toe walk due to spasticity is by botulinum toxin (BTX) injection into the calf muscles. Assessments of serial biopsies from the gastrocnemius in CP in humans comparing BTX with saline has as yet not been published, but injections into the tibialis anterior in the non-spastic rat has shown positive acute effects (Ateş and Yucesoy, 2014; Yucesoy et al., 2015). After 1 month, atrophy and decreased active force of all muscles in the anterior compartment of the lower leg was evident (Kaya et al.). There was an increased content of hydroxy-proline in the BTX group compared to rats injected with only saline. One key biomechanical finding of animal studies noteworthy of clinical testing is that the BTX group also showed elevated muscle passive forces (e.g., Kaya et al.). Another potentially functionally important biomechanical finding shown in animal studies was a decrease in length range of force production reported for muscles exposed to BTX (Kaya et al.; Yilmaz et al., 2021). Therefore, studies on what happens before and after botulinum toxin injections in the muscle in children with CP is highly warranted. A new method developed based on percutaneous muscle microbiopsies is promising (Corvelyn et al.), and can, with repeated biopsies during growth, hopefully in the future be used in understanding the natural history of muscles in CP. The gait may be improved if the calf muscle is lengthened by Achilles tendon lengthening, which will increase the relative length of the already long tendon even further. Here, there is a great risk of future weakness of the calf muscles if both legs are affected, as in spastic diplegia, and the child might develop crouch gait. A more conservative and safer operative method is to just release the gastrocnemius from the soleus fascia and sometimes also release the soleus aponeurosis. Few experimental studies have been conducted on what actually happens when an aponeurosis is cut (Brunner et al., 2000; Jaspers et al., 2002; Ateş et al., 2013). The mechanism of acute effects assessed computationally (Yucesoy and Huijing, 2009) showed that major sarcomere shortening in muscle part distal to a cut leads to a decreased muscle force, and overall increased sarcomere length heterogeneity causes muscle peak force production to move to a longer muscle length. Brunner et al. investigated aponeurotic release of the gastrocnemius in the rat. They found that the length of the muscle complex was increased, and the cut and resulting defect of the aponeurosis was filled with more compliant scar tissue (Rivares et al.). This is highly important since it suggests that the above mentioned acute aponeurotic release effects may also prevail after recovery, which needs to be tested in patients.

Regarding the force transmitting role of the epimysial connective tissue in patients with CP, active muscle length-force characteristics measured intraoperatively directly at the muscle tendon were shown to be affected by applying varying stretching on adjacent tissues of the tested spastic muscle (Smeulders et al., 2005). Likewise, mere distal tenotomy of the spastic flexor capri ulnaris muscle did not eliminate its contribution to the wrist flexion moment when epimuscular connective tissues were left intact (de Bruin et al., 2011), implying that the perimuscular connective tissues other than the proximal and distal tendon are important in transmitting force from a muscle to their target joints and structures. The role of collagen-reinforced connective tissues in the limited joint motion in spastic CP is still open to debate (de Bruin et al., 2013, 2014). Due to a highly variable distribution of intramuscular collagen content within muscle, the amount collagen content itself does not relate to the actual passive muscle properties during mechanical testing as was shown in mouse hind limb muscles (Binder-Markey et al.). Experimental work on rabbit lower leg muscles showed that passive load bearing properties of different muscles do vary. More importantly, mechanical testing of isolated muscle fibers compared, respectively, to bundles, fascicles, and whole muscle implied that the relative contribution to passive resistance changes from intracellular titin in isolated fibers to the extracellular perimysium in whole muscles (Binder-Markey et al.). However, *in vivo*, passive stiffness of monoarticular lower leg muscles was shown to be influenced by knee angle indicating intermuscular interactions may change muscular mechanics beyond a single muscle level. Note that, in CP patients, intraoperative testing of spastic semitendinosus and gracilis muscles did not show high passive forces measured at their tendon as a function of knee joint angle (Kaya et al., 2019, 2020). This suggests that in CP rather than the passive state, the active state is the determinant of the pathological condition, and co-activation of synergistic (Kaya et al., 2018) and also antagonistic muscles (Ateş et al., 2018b) was shown to elevate the active knee flexor force of individual spastic hamstrings significantly. Therefore, in the context of issues addressed in this Research Topic, mainly “the role of the muscle extracellular matrix and other myofascial tissues continuous with the epimysium on muscle force production” considering the possible influence of altered passive connective tissue structure and properties on the contractile apparatus in the muscle fiber and sarcomere level can provide the leverage to understand mechanisms of pathological conditions and effects of treatments for improved control over them.

Other degenerative conditions affecting muscle structure degrade gait, and early detection can help guide treatment. One example is myotonic dystrophy type 1 (the most common form of adult onset muscular dystrophy). Kim et al. have shown that functional gait assessments, such as the Functional Ambulatory Profile, correlate well with fat infiltration in lower extremity muscles and can serve as inexpensive early or longitudinal biomarkers in clinical studies.

Effects of blunt injury to the hindlimb muscles and to the lungs have been studied by Gihring et al. in lean vs. obese rats. They showed that obesity impairs the normal extracellular expansion up to 8 days past the injury, which is otherwise a normal part of the healing process. Obese rats, with fat deposits in the muscle, have a longer healing period with a prolonged period with increased pro-inflammatory cytokines and an altered satellite cell gene expression. This shows that the ECM buildup as a part of a healing process may be altered in different diseases probably not only in obesity, but also in, e.g., diabetes and maybe also in CP. Even lean children with CP have more signs of fat in the muscle compared to control when investigated with MRI (Noble et al., 2014).

## Author Contributions

All authors listed have made a substantial, direct, and intellectual contribution to the work and approved it for publication.

## Conflict of Interest

The authors declare that the research was conducted in the absence of any commercial or financial relationships that could be construed as a potential conflict of interest.

## Publisher's Note

All claims expressed in this article are solely those of the authors and do not necessarily represent those of their affiliated organizations, or those of the publisher, the editors and the reviewers. Any product that may be evaluated in this article, or claim that may be made by its manufacturer, is not guaranteed or endorsed by the publisher.
